# Oral Care Experiences of Latino Parents/Caregivers with Children with Autism and with Typically Developing Children

**DOI:** 10.3390/ijerph16162905

**Published:** 2019-08-14

**Authors:** Lucía I. Floríndez, Daniella C. Floríndez, Francesca M. Floríndez, Dominique H. Como, Elizabeth Pyatak, Lourdes Baezconde-Garbanati, Jose C. Polido, Sharon A. Cermak

**Affiliations:** 1 USC Mrs. T.H. Chan Division of Occupational Science and Occupational Therapy in the Herman Ostrow School of Dentistry, University of Southern California, Los Angeles, CA 90089, USA; 2 SOS Mentor, Los Angeles, CA 90089, USA; 3 Willamette Academy, Willamette University, Salem, OR 97301, USA; 4 Keck School of Medicine, University of Southern California, Los Angeles, CA 90089, USA; 5 Division of Dentistry, Children’s Hospital Los Angeles, Los Angeles, CA 90027, USA

**Keywords:** Latinos, oral care, health disparities, children, culture, autism spectrum disorder

## Abstract

As a result of various barriers, several pediatric populations are at risk for poor oral health, including children with disabilities and children from under-represented populations, such as Latinos. To this end, this study aimed to better understand the factors that affect the oral health experiences of 32 Latino parents/caregivers from 18 families (*n* = 8 with a typically developing child and *n* = 10 with a child with Autism). Using a qualitative descriptive methodology, each family was interviewed twice. Interviews were audio-recorded, transcribed verbatim, and coded thematically to identify the individual, social, systemic, and culturally rooted factors contributing to oral health disparities in the families. The three themes that arose were “Why would I want to start trouble?”: Latino parents’ dissatisfaction with dental treatments, costs, and fear of the dentist and health care providers because of their ethnic minority status as key factors inhibiting receipt of dental care; “We have to put our children first”: prioritizing the oral care activities of their children over their own individual oral care needs; and “We always keep baking soda around”: familial and cultural influences on oral care habits. Understanding the oral health beliefs and experiences of Latino parents and caregivers of children with and without autism is critical for developing targeted prevention and intervention programs and reducing oral health disparities.

## 1. Introduction

An important component of pediatric health care is oral care, which has a direct influence on health and quality of life [1]. Oral health related quality life measures the degree to which oral problems disrupt normal social functioning and lead to major changes in activities of daily living, such as the ability to work or attend school [2,3] and overall impact on emotional and functional well-being [4]. Oral health of children is of particular importance because children who experience early childhood caries are at higher risk for developing gingivitis, periodontal disease, and other dental problems as they age [5]. Dental care is a common unmet health need in U.S. children [6], associated with the highest financial barriers compared to other areas of health care [7].

Children who are from an under-represented minority population [8,9,10], from lower-income or lower-education households [11,12], or have a special health care need are at increased risk for experiencing oral health disparities [13,14]. Sociodemographic challenges can also further complicate the experience of raising a Latino child with a special health care need like Autism Spectrum Disorders (ASD) [15] and obtaining oral care [16]. Evidence suggests that race/ethnicity, socioeconomic status (SES), and poor oral health are related, such that Blacks, Latinos, and groups with low SES are at highest risk for caries, oral-related chronic diseases, and poorer overall health status [17,18,19]. Additionally, research has shown that children with ASD experience more barriers to oral care in the home and at the dentist and have less access to oral care than their typically developing peers [20].

In describing the oral health and dental needs of children with ASD, conflicting information exists pertaining to whether they have higher caries rates [13,21], comparable rates [22], or lower rates [23,24] compared to typically developing children. Despite lack of consensus regarding caries rates, there is general agreement that individuals with ASD display behaviors that increase their risk for poorer oral health outcomes [25]. The behavioral characteristics noted in ASD impact daily functioning in children with ASD, including completing routine oral care practices like tooth brushing and finding a dentist [20,26]. A number of studies have specifically examined the contribution of sensory factors to oral care challenges in children with ASD and have suggested that sensory factors exacerbate oral care challenges [20,27].

Studies focusing on oral care in Latino children with ASD have not specifically been addressed. However, this is a key area where more research is needed, as Latinos disproportionately experience oral health problems when compared to other ethnic groups [17,28]. Latinos have higher rates of dental caries, periodontal disease, and tooth loss than non-Latino white individuals [29,30,31]. Only 32.9 percent of Latino children obtain dental care in a year compared with 52.5 percent of white children, even after controlling for SES [32]. Additionally, of all race/ethnicities, Latino children have the highest likelihood of never having seen a dentist [33], the lowest average number of dental visits of all racial and ethnic groups [29], and the highest rate of untreated tooth decay and untreated caries [33].

Among Latinos, factors contributing to oral health disparities may include lack of access to care as created by structural and systemic barriers [34] and cultural and familial influences [35,36]. Culturally influenced factors that affect dental care utilization may include behaviors, beliefs, attitudes, and values, such as diet, level of acculturation, care of primary teeth, concern for oral health, and dental knowledge [37,38,39,40,41]. Research has shown that Latinos are less likely to believe in the need for regular professional dental care, more likely to have misperceptions about oral health, and less likely to have access to care than the general population [42].

Latinos and children with disabilities such as ASD are underrepresented populations that make up a sizable number of individuals in the U.S. Latinos are the largest and fastest growing minority group in the United States [43]. Similarly, the prevalence of children with ASD has been increasing, such that one in 59 [44] has Autism spectrum disorder. Despite representing sizeable numbers of the population, there is a shortage of current research documenting oral health practices of any Latino family units, with typically developing children or children with special health care needs, such as ASD. Thus, this qualitative study was designed to examine oral health attitudes, beliefs, and practices in Latino families with and without children with Autism.

## 2. Materials and Methods

### 2.1. Research Design

This article presents a sub corpus of findings from a mixed-methods study comparing the oral health beliefs, attitudes, and practices of Latino families of children with ASD to those with typically developing children. This qualitative, descriptive study presents findings from the larger data set designed to answer the following research question: How do Latino families with and without a child with ASD experience oral health?

The methods used were guided by the naturalistic inquiry approach outlined by Lincoln and Guba (1985) [45], where a qualitative descriptive process [46,47] was used to describe phenomena and present the details of daily life [48,49] of participants. The aim of the research was not to establish a generalizable body of knowledge or confirm a priori hypotheses about this population. Instead, this study emphasized exploring the participant’s individual experiences and the meaning they attribute to these experiences in a natural setting while uncovering common themes within and across participants. Semi-structured interviews were the primary form of data collection, with content drawn from extensive analysis of interview transcripts, audiotapes, and research field notes.

### 2.2. Participants

Eligibility criteria for participating families were (a) self-identify as Latino/a; (b) able to speak, read, and write in either English or Spanish; (c) parent/caregiver/family member of a child between ages six and twelve who is typically developing OR is diagnosed with Autism Spectrum Disorder; (d) use dental services in Los Angeles, and (e) live in the greater Los Angeles, California area. Participants were purposefully recruited through word of mouth, flyers, via social media posts in local parent support groups on Facebook and Instagram, and through partnerships with local community centers and occupational therapy clinics.

### 2.3. Procedures

All study documents were professionally translated into Spanish, including recruitment materials, consent forms, and demographic questionnaires. Prior to conducting study activities, ethical approval was granted by the University of Southern California Institutional Review Board (HS-16-00921). A semi-structured interview guide was created by the lead author and reviewed by all authors to ask families about dental care experiences and their perceptions about oral health. In line with qualitative descriptive methods, these questions were purposely kept open ended in order to prompt discussion and elicit natural answers from participants [50]. These questions were piloted with two Latino parents/caregivers to ensure clarity prior to using the interview guide in this study. (See Table 1 for the semi structured interview guide). Two bilingual study team members (the first two authors) with extensive interview experience conducted and audio-recorded the interviews with Latino parents/caregivers and their children/family members (in English (*n* = 22 interviews) or Spanish (*n* = 14 interviews), based on participant preference). In 10 of the 18 families, other family members, including the primary caregiver’s partner/spouse, or the child’s grandparents, cousins, or siblings, joined the primary caregiver in contributing to the interview answers. A total of 32 different family members and caregivers participated in the interviews, and each family completed two interviews, for a total of 36 meetings across the 18 families. Data collection took place between May and December 2017 in locations chosen by the families (e.g., the family home or homes of extended family members). Consent was obtained prior to the interviews, which each lasted 35–180 minutes. Participating families received $60 compensation for their time.

Each family also completed a demographic questionnaire and the Bidirectional acculturation scale for Hispanics (BAS) [51]. This scale measures bi-dimensional acculturation by asking about language-related behaviors, personal preferences, and social events that signifies the respondent’s level of both Latino and non-Latino cultural traits and has shown high reliability and validity among diverse groups of Hispanic/Latinos [51]. Acculturation is indicated by a low score (≤2.5) in the Hispanic domain paired with a high score (>2.5) in the non-Hispanic domain, and bi-culturation is defined as a high score (>2.5) in both domains [51]. Acculturation scores are reported in Table 2.

### 2.4. Data Analysis

All interviews were transcribed verbatim, and bilingual research members translated Spanish language transcripts into English for analysis. Rigor was obtained by employing triangulation and reflexivity as strategies for evaluating the quality and credibility of the data. To maximize reflexivity during this process, interviewers completed field notes after each interview, documenting their personal biases as they emerged. These notes allowed the researchers to reflect on their position within the context being studied and understand how these biases might be influencing the research process. Following Lincoln and Guba’s [45] criteria for establishing trustworthiness in qualitative research, triangulation via co-coding of emerging themes was done by the first two authors, who independently reviewed and coded the interviews. Using a thematic analysis approach particular to qualitative description studies [52], the first two authors coded the first four transcripts, and developed a preliminary set of codes to identify significant statements with reference to perceptions about the individual, social, and systemic factors contributing to oral care routines and overall oral health in the families. While coding, careful attention was given to both commonalities across participants’ experiences, as well as negative or disconfirming cases. Following this step, the entire research team met to discuss interpretation of and refine codes, further establishing credibility and dependability of the data via peer examination by multiple data analysts. Once the codebook was agreed upon by the research team, subsequent coding was done by research team members on NVivo 11 [53] using a constant comparative approach to examine overlap in coding [54]. The research team met bi-weekly to review the coded transcripts, discuss the coding process, and improve inter-rater reliability until consensus was reached about code definitions and overarching themes. Data collection and analysis occurred simultaneously, and coding continued until data saturation was achieved.

## 3. Results

### 3.1. Sample Characteristics

Participants were 32 Latino parents/caregivers from 18 Latino families (eight families with children with Autism Spectrum Disorders (ASD) and 10 families with typically developing (TD) children). Initially, equal samples were recruited from families with TD children and families with children with ASD. However, two families from the ASD group became unable to participate in the study for an extended time, and we recruited two more families to participate, increasing the sample size in the ASD group from eight to ten families. Then, the two families who appeared lost to follow up from the ASD group re-established contact with the study team and were interviewed for a second time as initially planned.

Table 2 summarizes the sample characteristics derived from a demographic questionnaire completed by the primary caregiver.

Notably, the sample represented a bicultural group of participants, with parents/caregivers in both the TD group and the ASD group scoring over 2.5 in both Hispanic and non-Hispanic domains. The sample is also representative of various types of Latinidad, as families were from varied countries of origin, including families from Central and South America.

When presenting the findings from the interviews, participants are identified by pseudonyms, and minor changes in details are used to protect their identity. All quotes are presented as the English translation for ease of reading. The three themes related to oral care experiences that arose from the interviews include “Why would I want to start problems?” (Vulnerability and mistrust of providers), “We have to put our children first” (Parents prioritizing the oral care activities of their children), and “We always keep baking soda around” (Culturally and family informed oral care practices).

### 3.2. Findings

#### 3.2.1. “Why Would I Want to Start Problems?” (Vulnerability and Mistrust of Providers)

Latino families’ feelings of vulnerability and mistrust in healthcare providers was a theme that was widely evidenced by the content discussed in interviews due to participants status as an ethnic minority and, in some cases, their immigration status. Some families worried that going to a healthcare visit would result in being identified as an undocumented immigrant and potentially even being turned over to immigration officials. For example, Celia compared going to dental care visits in the United States to “walking into the arms of *La Migra”* (immigration and customs enforcement agents), because she did not trust that healthcare workers would not betray her immigration status. Valeria also admitted that her in-laws “rarely went to the dentist in this country [USA] because they don’t have papers,” which was common to hear from participating families. Armando echoed this sentiment by explaining that he only went to the dentist when he visited Mexico, because “those are the only doctors there that I trust.”

Araceli spoke about feeling traumatized and misled by her dentist when she received dental care shortly after arriving to the United States,

“In Mexico, a horse kicked me in the mouth and left my teeth loose. When I first came [to the United States], I was determined to fix my teeth. So I began to work and save all my money, and I went to a dentist. I don’t remember his name, but I think he was a new dentist who was still learning. So I went to him, and he told me he had to cut out my root to fix my tooth...and he ended up cutting a piece of my muscle…rather, he cut a chunk out of my mouth. Then, he told me he would fix it by putting in something in behind my teeth. But how could he put anything else in when he had already cut out and removed that section? I left feeling very traumatized with what he did to me, and since then, I have not returned to the dentist.”

As a result of her experience, Araceli resorted to extreme measures. In order to compensate for having part of her mouth cavity missing and loose teeth, she used glue to hold her teeth in her mouth. She explained, “You know what I have to do to keep my teeth in the right way, to this day? Don’t laugh…I have to use Krazy Glue on my teeth.”

Araceli further articulated the vulnerability she felt because, despite the dentist malpractice, he still required her to pay thousands of dollars for the services rendered. She chose not to complain or pursue legal action against him, because he threatened to use her immigration status against her. She continued, “Despite what he did to me, he knows I don’t have papers and said he could have me deported…and well, that matters more…why would I want to start problems?”.

#### 3.2.2. “We Have to Put Our Children First” (Parents Prioritizing the Oral Care Activities of Their Children)

Study participants often framed the importance of promoting the oral care activities of their children over their own individual oral care needs. Notably, this theme seems to be evident in that, despite the barriers and limitations that make self-care and oral health activities difficult, parents prioritize and do the best they can to promote their children’s oral health. Paola noted, “I think that we Latinos don’t take care of ourselves…we don’t brush our teeth or see the dentist because it is too expensive, and we have to put our children first.” This theme about parents putting the needs of their children first was echoed among other participants, including Violeta, who said, “Everything that deals with me is less important than the kids are, as long as they are taken care of. My family comes first.” Estefania relayed,

“I have problems with a tooth, and they told me I have significant bone loss…maybe because I am prediabetic, like my mother and grandmother are diabetics…and I still have problems with this tooth, problems, problems, problems, but the truth is here it is too expensive to get help, and I don’t have dental insurance. But, I make sure I take my boys to the dentist, so they don’t get problems with their teeth too.”

Isabella shared that while she recognized that her actions of putting the needs of her children first often meant that she did not meet her own health needs, she felt it was the only way she could care for them. What’s more, she felt like it was her duty to go without in order for her children to maintain their oral health, “I buy them [her sons] salmon, because it’s supposed to be good for their teeth. And it’s expensive. So if there is enough for me, I eat it. But I try to eat when I’m working [cleaning houses], so that I don’t take the food from my boys. That’s what a mother does.”

Caregivers expressed that they learned about priorities pertaining to oral care routines and putting the needs of their children first from their own parents. Araceli spoke of her own childhood growing up in Central America,

“The truth is, when I was growing up, we did not brush our teeth; we did not even have a toothbrush. Instead, we would rinse our mouths. Toothbrushes did not even exist; actually, they existed, but only for people that had money, not for the poor people. Can you imagine if my mother had to buy toothbrushes for all of us? There would not be enough money. There was only enough money to eat, and there were other priorities for the family like eating, buying school uniforms, and staying fed.”

#### 3.2.3. “We Always Keep Baking Soda Around” (Familial and Culturally Informed Oral Care Practices)

Participants also spoke of American cultural practices and expectations in relation to oral care that were different from those from their country of origin, and how the medical system in the United States was difficult to navigate. These ingrained previous experiences influenced their beliefs and practices once in the United States.

Estefania shared how her husband grew up in rural Mexico and had very poor oral health habits because of growing up in poverty with absent parents. Because of his childhood experiences, her husband grew up to be fearful of the dentist, doubting that he would receive help. She described how the only dental attention her husband received in his life was from a mobile dental clinic,

“He maybe went to the dentist once in his life growing up in Mexico, and it was only because a truck drove through his neighborhood when he was a child. It was from the city, and it was a mobile unit, a Mobile Dentist. But there, it would only drive by maybe every 3 years. And so he came out running and they told him, ‘we can’t help you, we need to talk to your mother.’ And well, his mom was not around. So instead, he asked a neighbor, ‘please tell them I’m your nephew, give me your hand, so then they will help me. Look, it hurts.’ And I think that was the only time he got a cleaning as a child. And the dentists told him “you need a lot of help,” but he never got it. So then he stopped trying, because he did not think that they would help him.”

When speaking about her mother-in-law who came to the United States from Central America, Jennifer stated that in Central America,

“It’s a two-hour bus drive and then it’s another four-hour donkey ride to get to her home…it’s that remote. And that translates into how she was raised regarding the importance of dental care. Was it important? Like, literally they didn’t have running water. They didn’t have any electricity. It’s a different atmosphere completely. So I’m sure that translated in how she raised her boys here in America, because it’s like, ‘Well, if you do brush your teeth, that’s great because it is healthy to do it. But if it doesn’t happen, it doesn’t happen.’ You know, that’s not the highest priority on the list.”

Additionally, participants spoke about the difficulty in navigating the dental care system and using public insurance in the United States. Elisa shared,

“My half-sister, she lives in El Salvador. When she came, she had a lot of cavities, so I was flossing, I was brushing her teeth. I was taking her to the dentist. And she had Medi-Cal, but with Medi-Cal, they just do one tooth at a time. So I was taking her to her appointments, but I’m like, how do people do it? Because if they’re just seeing you for one tooth, and then that’s it, next appointment in a month. So you have to go back for each tooth, you know? Oh my god, it’s too much.”

Influence about oral care also directly came from family and social connections. Isabella described her mother’s influence on her decisions about taking her child in for dental care,

“I first took [my child] to the dentist he was nine months old. The doctor said, ‘You know, he’s going to need to be checked,’ so I took him. And then my mom said, ‘Why? I never took you guys, kids don’t need it. The first teeth are going to fall out either way.’ So that was in my head. I took him, but then I was like, ‘I’m not going to do the follow up, because the teeth are still going to come off either way.’”

In a similar story about how seemingly conflicting values and beliefs about oral care were passed on within families, Valentina discussed learning about the value of keeping her teeth healthy from her South American parents:

“My parents said, ‘You have to take care of your teeth, because your own teeth are better to have than dentures.’ I remember my parents would tell me that, and luckily, maybe it’s hereditary, but I have good teeth, and I also know it’s because I took care of them all my life. My mom did too. My dad, not so much. When he was younger, he lost a lot of his own teeth, and then he had to get dentures, and then implants. He had a lot of trouble because he didn’t take care of his teeth.”

Parents also learned culturally rooted remedies and methods for performing in-home oral care routines and addressing oral problems from their own parents. In Isabella’s home, she used several home remedies, including diet changes: “I try to get them to eat strawberries too, because I heard that they help with their teeth.” She also mentioned baking soda as an alternate brushing option: “When my gums were sensitive my mom actually told me to put a little bit of lemon and baking soda and just leave it on the gums just in case…We always keep baking soda around.” Using baking soda was supported by many parents as something they were taught to use in place of toothpaste, because toothpaste was too expensive. Mia shared why her mother encouraged her to use baking soda, “Growing up, all we did was rinse with bicarbonate…that’s what I used my whole life. We knew it cleaned things because it was what you used to clean in the houses of the rich people…it’s called Baking Soda.” Other suggestions that came up were using cloves, as mentioned by Estefania: “[My mother] told me to put cloves on the tooth that hurt, and I would chew it, and that it would help improve the pain. She learned that from her mother.”

## 4. Discussion

This qualitative study examining oral care practices in Latino families provides a rich picture of factors contributing to oral health disparities among Latino children with and without disabilities. A significant finding of the study was the way that study participants embodied a shared Latino identity and cultural experience, and how factors like immigration status and value of family, impacted their experiences with oral care. Study participants discussed factors related to levels of acculturation, mistrust of healthcare providers from the United States health system and overcoming barriers in order to provide adequate oral health care for their children. The findings of this study not only grow the current body of research, but also add new information that expands our knowledge of culturally relevant ways to address oral care disparities in Latinos.

First, like previous research, the findings of this study confirm established barriers to oral health care for Latinos. These barriers include systemic variables such as cost and lack of insurance that impact access to oral care [34,37,55], the ability of patients to navigate the healthcare system and find personable dentists [56], lack of knowledge about oral health [57], and the impact of immigration status on receiving care [58,59,60]. Our study population consisted of participants from mixed immigration status. However, they were all very aware of how immigration status impacted their lives directly, or indirectly via friends or family members who reported difficulties with health care providers, or fear of being deported while at health care visits. Indeed, research has shown that noncitizens had a lower rate of dental service use, and higher rate of tooth extraction compared with US-born citizens [61], and higher rates of mistrust in the health care system [62,63,64]. Accordingly, the participants and their extended families faced financial barriers including a lack of insurance and high cost of dental treatment, and also had difficulty accessing care due to their immigration status.

Second, our findings confirm a narrative in Latino identity and culture pertaining to the importance of family, or *familismo.* Generally understood as the importance of immediate and extended family [65], *familismo* means family is highly valued, and these personal interactions within social networks influence care and actions related to health. Maupome et al. [35] explored the ties between *familismo* and oral care in a group of Mexican-Americans and noted that acculturation was associated with lower odds of discussing oral health issues, and participants preferred to discuss oral care with family members. In our study, parents framed the concept of putting the needs of family and children first as their “duty”, and something that was expected of them as parents, which aligns with other research that has identified the key roles that parents play in providing basic needs for their children [66,67], especially among mothers [68,69]. However, despite the best efforts of parents to put their children first, oral disparities persisted due to the numerous factors working against them. These findings also align with previous research on Latino families, which show the great lengths that parents go to prioritize their children’s health and wellbeing, sometimes at the cost of their own [70,71].

Finally, this study added new and unexpected findings to the sparse literature on Latino oral health care for children with and without disabilities by showing how Latino identity was a stronger influence in the families’ oral health experiences than the child’s disability status. At the onset of the study, we expected that Latino identity and culture would intersect with disability such that the ASD and TD families’ experiences would greatly differ, almost as parallel cultures, one being Latino, and one being disability culture. To be clear, the families’ experiences did differ in several ways based upon the child’s disability status, and parents shared some stories about oral care activities or routines that were more difficult for the children with ASD due to their sensory sensitivities. However, this paper presents unexpected findings: The families often spoke about their Latino identity and cultural values in a way that transcended the child’s disability status. Though the families had different life circumstances, families with and without a child with Autism shared similar perspectives related to their Latino identity and oral health. For example, undocumented parents in both the TD group or ASD spoke about being fearful about immigration issues surrounding obtaining dental care; this fear was not altered by whether the child had autism or not. Previous research with children with ASD indicates that in-home oral care activities and dental clinic visits are more challenging due to ASD related and sensory sensitivity symptomology [20,72]. We were therefore surprised at the prominent role that Latino identity and culture, even over the child’s disability, played in our qualitative findings. This finding has important implications for future research as well as intervention programs, namely that focusing on children with ASD as a whole may not be sufficient; rather, culturally-informed oral care interventions are critical for addressing Latino families’ needs [73,74,75]. Indeed, recent research has shown that employing a tailored approach to conducting interventions for Latino families that incorporates Latino cultural components are successful [76]. Understanding the importance of social and cultural influences on Latino patients’ health beliefs and behaviors and considering how these factors interact at multiple levels of the health care delivery system, will assist in devising interventions that take these issues into account to assure quality health care delivery to diverse patient populations [76,77,78], with a goal of reducing oral health disparities.

### Limitations

This study was a descriptive, qualitative study with a small sample of participants recruited from one geographic location in Southern California. The findings presented are therefore preliminary and cannot be generalized to Latino families outside of this study or in other parts of the country. Qualitative methods do not use statistical methods to make inferences about a population; rather, they are best for providing an understanding of the factors, contexts, and socio-cultural processes of understudied phenomena, such as the oral care beliefs and practices of Latino families with and without children with ASD. However, the Latino population is heterogeneous, with many different beliefs and perceptions. Therefore, given the relatively small sample size, future studies could examine the oral care experiences and practices of different groups of Latinos, include a comparison group in the interviews, and incorporate other methods for evaluating oral care experiences, such as a survey or undergoing a dental exam. Quantitative methods are ideal for surveying large groups of people and could be an effective next step to explore how these issues impact a larger sample of people to make more generalizable conclusions about the population. Additional limitations include lacking medical data about the children with ASD, such as no confirmed diagnosis and no information about severity of symptoms, which may have affected family responses. Lastly, the study was not a longitudinal design, and prolonged engagement with participants could be helpful to see how these processes unfold over time. Despite its limitations, this study contributes formative data on perspectives of Latino parents of children with and without disabilities regarding their oral care experiences and bringing attention to this under-researched and under-served community.

## 5. Conclusions

The focus of this study was to explore the oral care experiences of a sample of Latino families. Study findings confirm the barriers to obtaining oral care in the population and reveal the ways that Latino identity and culture influenced oral care experiences and beliefs in Latino families with and without children with ASD in similar ways. It became evident that the embedded values of the Latino participants were a prominent theme that influenced how they spoke about oral health and how they practiced oral care routines in the United States, irrespective of their having a child with ASD. Researchers and practitioners should consider the cultural implications of working with Latino families and be sensitive to their experiences. For example, to provide parents with the necessary tools and information to help their children, dental service providers need to understand how the oral care experiences of Latino families influences their service utilization. Further research is necessary to better understand how attitudes about disability affect oral care, as well as best methods to employ during intervention programs targeted for this population. 

## Figures and Tables

**Table 1:** Interview guide questions.

Guiding Question
Daily Routines
Tell me what your typical oral care routines are like?
Are there other people or things that influence your oral care/routines?
Is there any other information you would like to share with me about your oral health routines?
Perceptions of oral care
How would you describe your oral health status? (you, your child)
How do your experiences impact your child?
Is there anything that makes your oral care easier? Harder?
Cultural Background
How has your oral care changed, if at all, since you have been in the US?
How does your family background/beliefs/culture impact your oral health?
What are your beliefs about oral health? Where did you learn them?

**Table 2:** Sample characteristics.

Characteristics	Parents of cASD (*n* = 10)	Parents of TD Children (*n* = 8)
Language Spoken		
English	70% (7)	50% (4)
Spanish	30% (3)	50% (4)
Primary Caregiver Gender		
Female	90% (9)	100% (8)
Male	10% (1)	0
Years of Education (range) of participating parent	13 (6–19)	13.9 (7–17)
Mean Number of Children in family (range)	2.25 (1–4)	1.9 (1–3)
Age of child enrolled in study	9.0 (6–12)	7.9 (6–12)
Parent Nativity		
Central America (Mexico, Guatemala, El Salvador)	40% (4)	62.5% (5)
South America	10% (1)	12.5% (1)
United States	50% (5)	25% (2)
Acculturation*	* on a 5-point scale, scores over 2.5 indicate higher level of adherence to cultural domain. 2.5 of above in both categories indicates biculturalism	
Hispanic	2.958 (1.07)	2.979 (0.955)
Non-Hispanic	3.367 (0.641)	2.712 (1.024)

Data are presented as mean (range), percentage (*n*), or mean (standard deviation).
